# Decrease in wind stress leads to an increase in the above ground morphology and number of seeds of an invasive alien species, *Bidens pilosa* (Asteraceae)

**DOI:** 10.3389/fpls.2024.1445437

**Published:** 2024-11-08

**Authors:** Masayuki Shiba, Nagisa Kobayashi, Shiori Harada, Tatsuya Fukuda

**Affiliations:** ^1^ Graduate School of Integrative Science and Engineering, Tokyo City University, Tokyo, Japan; ^2^ Department of Natural Sciences, Faculty of Knowledge Engineering, Tokyo City University, Tokyo, Japan

**Keywords:** alien species, *Bidens pilosa* var. *pilosa*, morphology, seed production, wind stress

## Abstract

We conducted comparative analyses using an open-top chamber (OTC) to reduce wind stress to clarify the impact of decreased wind stress on the invasive species *Bidens pilosa* L. (Asteraceae), which ranks among the worst 100 species on the Invasive Alien Species List in Japan. Morphological analyses revealed that the number and size of leaves in the OTC group were significantly higher than those in the control group (wind). There was also a significantly higher investment in stems in the former than in the latter. No significant differences were observed in root dry mass; however, the resource allocation ratio to the roots was significantly higher in the wind group than in the OTC group. Although the total seed mass was greater in the OTC group, there were no significant differences in the ratio of resource allocation to seeds between the groups, and no significant differences were observed in the mass of each seed. However, the number of seeds was significantly higher in the OTC group. Adaptive changes in the leaves, stems, and roots to avoid and/or resist wind were reflected in differences in the number of seeds. In addition, a decrease in wind stress contributed to an increase in the number of seeds in *B. pilosa*. Such mechanisms are likely widespread because *B. pilosa* is often highly abundant in urban systems.

## 1 Introduction

Wind is one of the most ubiquitous environmental stressors that can strongly affect the development, growth, pollination, seed dispersal, insect herbivory, and reproductive yield of plants (Wilcock and Neiland, 2002; Hodges et al., 2004; Fernandez and Hilker, 2007; Turbill, 2008; Shiba et al., 2022c; Takizawa et al., 2022, 2023). Wind is an important factor at many levels in ecosystems, and avoiding wind stress using flexible and easily reconfigurable structures is an alternative strategy (Anten et al., 2009). How plants adapt to or acclimate to variable external forces depends on the intensity and frequency of stress and the plant structure.

**Figure 1:**
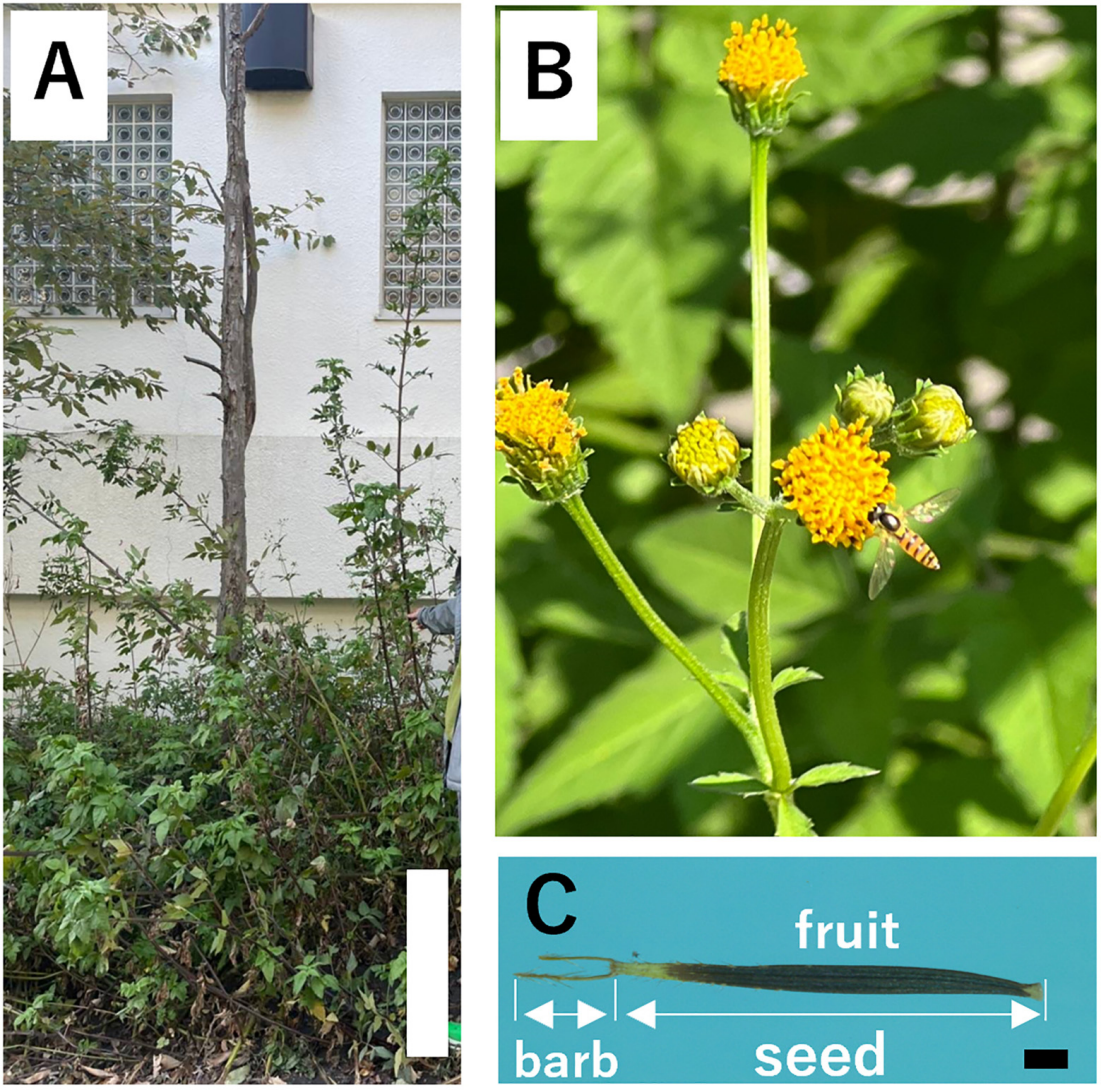
*Bidens pilosa var. pilosa* (bar = 50 cm) **(A)**. Inflorescences **(B)** and seed (bar = 1 mm) **(C)**.

Invasive alien plant species pose a serious threat to local biodiversity, ecosystem services, and environmental quality by replacing regionally unique native species with common nonnative species (Godefroid, 2001; Wania et al., 2006; Shochat et al., 2010; Rai and Singh, 2020). *Bidens pilosa* L. is an invasive plant in Japan and an annual species of herbaceous plants in the family Asteraceae that grows to approximately two meters tall (**Figure 1**). This species has pinnate leaves, heads consisting of approximately four or five broad white ray florets with many tubular yellow disc florets, and barbed fruits approximately 1 cm long. It is native to America but has expanded as an introduced species in other regions worldwide, including Eurasia, Africa, Australia, South America, and the Pacific Islands. It is widely used as a folk medicine in African and South American countries and is used to treat malaria (Adia et al., 2014), enteritis, nephritis, diarrhea (Chih et al., 1995), and gastric disorders, including peptic ulcers (Alvarez et al., 1999), antimalarial activity (Oliveira et al., 2004; Tobinaga et al., 2009), anti-inflammatory, anti-allergic, immunosuppressive activity (Pereira et al., 1999; Horiuchi and Seyama, 2006), antibacterial activity (Ashafa and Afolayan, 2009; Dagawal and Ghorpade, 2011), anti-leukemia activity (Chang et al., 2001), hypoglycemic activity (Ubillas et al., 2000), hepatoprotective effect (Yuan et al., 2008), and anticancer activity (Sundararajan et al., 2006). *B. pilosa* grows aggressively in cultivated fields, roadsides, and disturbed lands in the urban areas of Honshu, Kyushu, and Ryukyu, Japan, and often becomes weedy (Koyama, 1995; Asami et al., 1999). For example, in Japan, it has been reported that dragonflies become unable to move after their wings become stuck in the achenes of *B. pilosa*, resulting in their deaths (Fukasawa and Takahashi, 2017). The majority of the flying insects found on the achenes of *B. pilosa* were from the family Odonata, which were particularly frequently found was considered to be that their thin wings make it easy for them to pierce the achenes, and that many of them had the habit of using the tips of herbaceous stems as perches (Fukasawa and Takahashi, 2017). They use these perches not only to rest, but also to regulate their body temperature because they are cold-blooded animals that need to be exposed to sunlight to heat up during low temperatures, and they often rest in high places such as the tips of stems to heat up efficiently (e.g., Iwasaki et al., 2009). One of the reasons why many dragonflies found themselves on the achenes at high points of *B. pilosa* was that these places were easily exposed to sunlight. In addition, because *B. pilosa* inhibits the growth of other plants through allelopathy (Uraguchi et al., 2003), *B. pilosa* tends to be the only plant available for perching in the vicinity, which may increase the possibility of dragonflies becoming entangled in the achenes and dying. Therefore, the Ministry of the Environment has placed *B. pilosa* among the worst 100 species on the Invasive Alien Species List of Japan and urged caution against its growth. However, the barbed awns of fruits are stuck in the clothing, fur, fleeces, and socks of people who brush against this species and are dispersed by people (anthropochory), expanding their distribution in various parts of Japan.

With respect to seed germination after dispersal of *B. pilosa*, Chauhan et al. (2019) reported that germination was stimulated by light. Thus, its emergence was the greatest on the soil surface, indicating that seeds transported by people can easily germinate anywhere. Moreover, previous studies on the effects of this species on the environment indicate that dispersing seeds into cropping fields is becoming widely recognized as a major impediment to economic food production (Reddy and Singh, 1992). Even more concerning is that some biotypes of *B. pilosa* have acquired herbicide resistance. For example, López-Ovejero et al. (2006) reported that acetolactate synthase (ALS)-inhibiting herbicide-resistant biotypes of *B. pilosa* in Brazil and other biotypes in Mexico, were resistant to several herbicides, including glyphosate and paraquat (Alcántara-de la Cruz et al., 2016). The explosive spread of *B. pilosa* in urban cities may help it spread to various areas, including cropping fields, through seeds attached to people’s clothes. Therefore, an urgent examination of the reproductive strategies of *B. pilosa* in urban environments is important for developing an effective integrated management program for this species. In the present study, we examined the reverse chain of cause and effect and investigated how wind patterns alter *B. pilosa* productivity.

**Figure 2:**
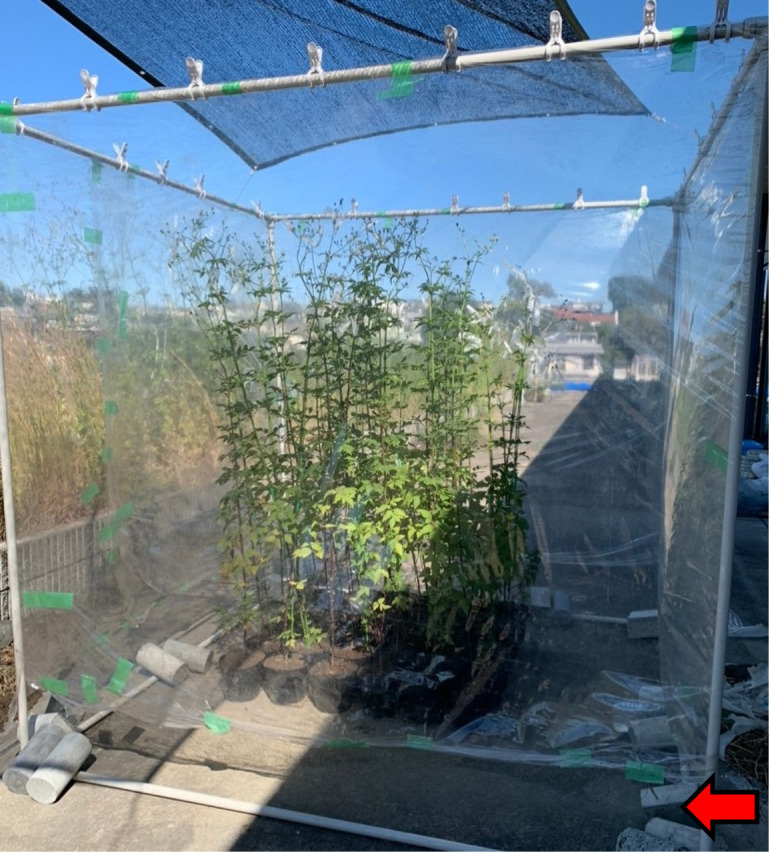
*B*. *pilosa* being grown in a self-made OTC. The red arrow indicates the air inlet.

Which system best compares plant impacts on wind stress reduction? Experiments on the effects of wind on plant growth have been conducted using fans, wind tunnels, and artificial shelters (Cleugh et al., 1998). However, few field experiments have manipulated wind to determine its effects on wild plant growth (Bang et al., 2010). We selected a windbreak design to limit the impact of shading and enable direct measurement of the wind effect, although some wind turbulence is inevitable when constructing wind barriers (Moen, 1974). Open-top chambers (OTC) were first used to study the effects of air pollution and were later adapted for elevated CO_2_ research (Maggio et al., 2009), They are often used to warm aboveground systems, especially in Arctic and alpine ecosystems (Walker et al., 2006). However, as the temperature inside an OTC increases, it is necessary to consider the effects of temperature and wind. Bang et al. (2010) reported that leaving a 15–20 cm opening near the ground without a roof when protected by windbreak shields could prevent the greenhouse effect. This simple design is ideal for examining the effects of wind stress on plants. Therefore, we established an OTC (**Figure 2**) and conducted an experiment to compare the growth of *B. pilosa*. This comparison revealed the effect of wind on the growth and the morphology of *B. pilosa* in an environment with reduced wind stress. In addition to them, seed production plays a major role in determining the final plant fitness and is an important factor in the ecology and evolution of plant life histories (Donohue, 2005; Dwyer and Erickson, 2016; Ishii et al., 2022; Marui et al., 2023). Thus, consideration of the reproduction of invasive species in Japan requires attention to seed production. Many studies have been conducted on the number and size of seeds in seed production (e.g., LePage et al., 2000; Moles and Westoby, 2004; Jones and Muller-Landau, 2008; Muller-Landau et al., 2008). Qiu et al. (2021) reported that seed production varies depending on the size of the individual, and therefore the number of seeds can vary greatly within a species (Qiu et al., 2022), indicating that it is necessary to analyze the differences in seed production of *B. pilosa*. This study aimed to clarify the effects of stress reduction on the growth and production of *B. pilosa* using OTC.

## 2 Materials and methods

### 2.1 Cultivation conditions

Sprouts of *Bidens pilosa* var. *pilosa* was confirmed on April 18, 2022, in a hedge on the campus of Tokyo City University, and the plants were successively replanted in polypods (7.5 cm diameter, 220 mL volume) on May 26, when the true leaves were observed to have developed. Individuals collected at this site were confirmed to have grown and fruited with the parental individuals in the previous year. For cultivation, black soil was used, and plants were grown in polypods (7.5 cm diameter, 220 mL volume) and replanted into 12-cm (830 mL volume) and 18-cm (2900 mL volume) diameter pots when roots started to grow through the drainage holes to avoid root clogging during plant growth. Before the cultivation experiment, 36 *B. pilosa* var. *pilosa* plants grown in OTC without direct wind protection (hereafter referred to as the ‘OTC group’) and 36 plants grown without wind protection (hereafter referred to as the ‘wind group’) were sorted to ensure that individual size was not biased. In the OTC group, one individual died during cultivation, so the final number of plants was 35.

The cultivation experiment was conducted on June 20 on the rooftop of Building 6 of Tokyo City University. The plants in both groups were arranged in a 6 × 6 configuration. In this environment, wind speeds greater than 6 m/s were recorded using a handheld anemometer on cloudy days. The OTC group was not directly exposed to wind because the anemometer propeller did not rotate. However, an air inlet approximately the size of a pot is left open to eliminate the greenhouse effect. Although the outer pots are somewhat more exposed to external boundary effects than the inner pots, they are not directly subjected to wind loads by the plant body, which is not considered to be within the range of influence for this study. When typhoons passed, the plants were temporarily evacuated indoors to avoid damage from overtopping by strong winds. Watering during cultivation was performed until the water pool was filled at least once a day. Watering was not performed on days when the soil surface was not dry enough to prevent root rot.

**Figure 3:**
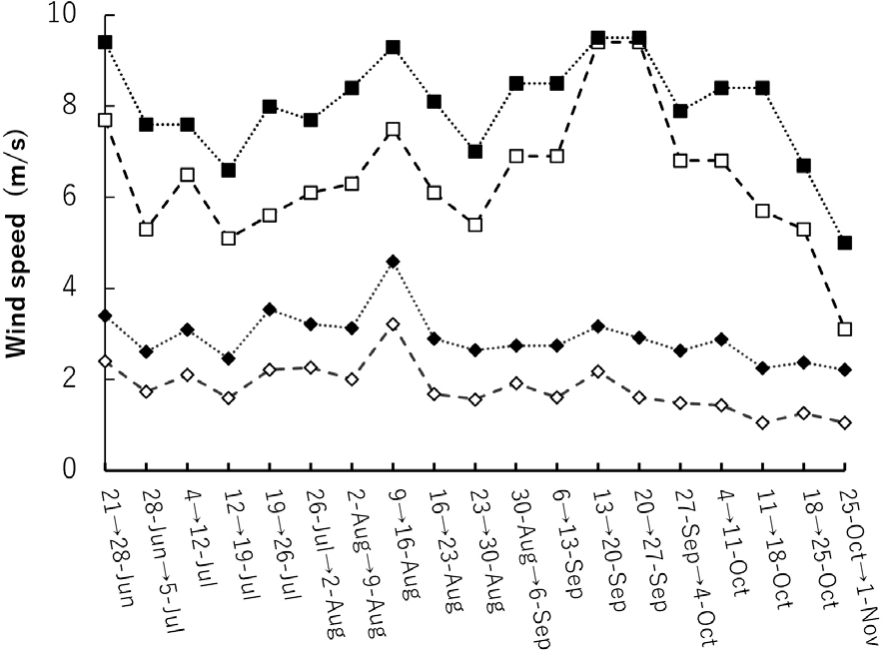
Wind speeds during the experimental period near the cultivation site. The data used were AMeDAS data at year 2022. Closed solid indicates the maximum weekly wind speed in Tokyo and open solid indicates the maximum weekly wind speed in Fuchu. Closed rhombus indicates the weekly average wind speed in Tokyo and open rhombus indicates the weekly average wind speed in Fuchu.

For wind speed data during the cultivation period, historical weather data recorded by the Automated Meteorological Data Acquisition System (AMeDAS) set up by the Japan Meteorological Agency was used. AMeDAS data from nearby Fuchu and Tokyo were used as wind-speed data during the growing season. For wind speed data, the average and maximum wind speeds during a week were used for the period from June 21 to November 1 (**Figure 3**).

### 2.2 Open-top chamber

To reduce wind stress, a self-made OTC was created; the OTC was covered with horticultural plastic sheeting (0.1 mm × 1.85 × 5 m) on the lateral side of the frame and fixed to the floor with the canopy open. An air inlet for circulation was installed at a height that did not exceed the pot size in front of the floor to eliminate the greenhouse effect inside the OTC, as described by Bang et al. (2010). The air inlet eventually reached a height of approximately 15 cm.

The reduction in radiation and changes in spectral composition near the chamber walls of the OTC could affect plant production and morphology (Hollister et al., 2023). Some studies have reported that the chamber wall reduces photosynthetic photon flux density by more than 10% (Drake et al., 1989; Nakashima et al., 2018; May et al., 2022; Hollister et al., 2023). However, because the OTC is open at the top, the photosynthetic photon flux density reduces the light the most in the morning and evening when the sun is at a low angle. However, around noon, the rate of its reduction by the chamber walls becomes very small because sunlight directly reaches the plants inside the OTC.

### 2.3 Growth rate measurements of above ground morphology.

Stem and leaf measurements were performed to determine the morphogenetic patterns of the aboveground parts as they grew under different wind stress environments.

Node counts were used as indicators of leafing, and the number of nodes per individual was counted. The measurements were performed once a week between June 21 and November 2, 2022. Three fully expanded leaves, starting from the uppermost leaf, were selected and photographed to determine the leaf area. This was analyzed using “ImageJ” image analysis software, and the average of three values was calculated. The leaf area was measured once a week between July 8 and August 26, 2022. On September 16, inflorescences began to form, and leaf development decreased.

Stem length and diameter at the base were measured using digital calipers (CD-15APX; Mitutoyo, Japan). The stem diameter was measured three times, and the average of the three measurements was used as the representative value for each individual.

The growth rate of one individual was calculated using the following equation:


$$G\left[\text{cm}/\text{days}\right]=\frac{l_{week}-l}{7\;}$$

where *G* is the growth rate of elongation per day divided by the weekly elongation of the stem divided by seven days, *l* is the stem length on the day of measurement, and *l*_week_ is the stem length seven days after *l*.

The number of inflorescences per individual was measured between September 27 and December 6 The data from November 11, when the highest number of inflorescences developed in both groups, were compared.

Seeds were harvested by wrapping the fruits in nets to prevent seed loss from inflorescences. The number of seeds per individual and average seed mass were measured for the harvested seeds.

After November 1, morphometric measurements were terminated because the stems and leaves had stopped growing, and dead leaves were collected from November 1 onward. December 22, the absence of new organogenesis was confirmed, and the Biomass analysis was initiated.

### 2.4 Biomass allocation

After cultivation, the plants were cut into roots, stems, and leaves and dried in a dryer (DRM420DD; Advantec, Japan) set at 100°C for at least 72 h. After removing the dried samples from the dryer, dry mass was immediately measured using an electronic balance (ATX224R; Shimadzu, Japan). The masses of naturally dried seeds were also combined to calculate the resource-partitioning ratio of the roots, stems, leaves, and seeds in one individual using the following equation:


$$\left(\text{Biomass allocation to each organ}\left[\%\right]\right)=\frac{\text{Dry mass of each organ }\left[\text{g}\right]}{\left(\text{Total dry mass of all organs}\left[\text{g}\right]\right)}\times 100$$

### 2.5 Statistical analysis

The R software was used for statistical analysis (R Core Team, 2024). After equal variances were obtained by comparing each measured item (F-test), t-tests were performed under the assumption of “equal variances” or “not equal variances.” The Student’s t-test was used when equal variances could be assumed, and Welch’s t-test was used when equal variances could not be assumed. For the comparison of Biomass allocation, which is a comparison of three or more groups, the Tukey–Kramer test was used.

## 3 Results

The results of the F-test analysis of variance showed that equal variances can be assumed for the number of inflorescences, number of seeds per individual, mass per seed, total seed mass per individual, dry mass of roots, and dry mass ratio above/underground, whereas equal variances cannot be assumed for the dry mass of stems and leaves.

### 3.1 Results of aboveground morphological measurements

**Figure 4:**
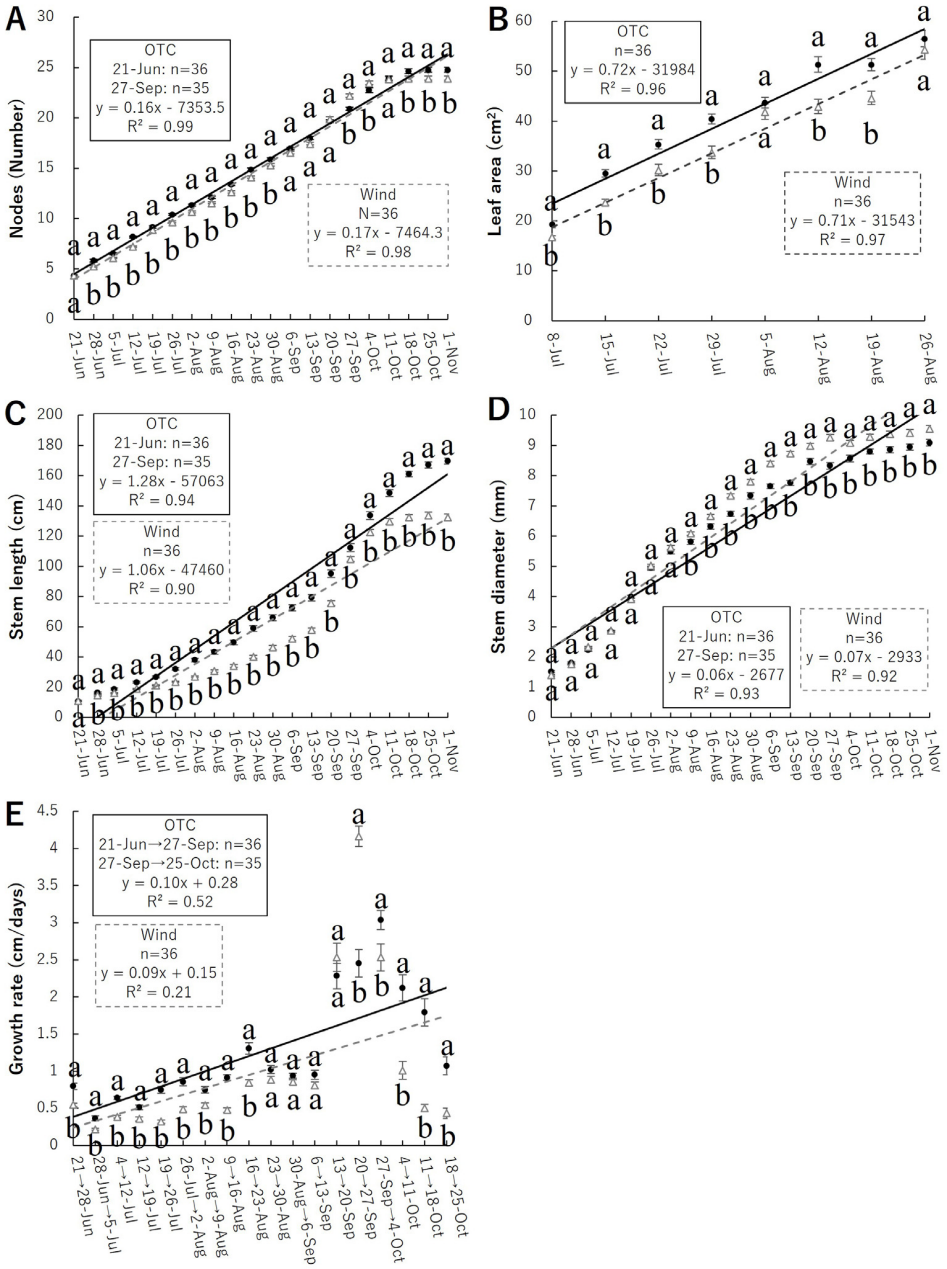
Relationship of morphological results; number of nodes **(A)** and leaf area **(B)**, stem length **(C)**, stem diameter **(D)**, growth rate **(E)**; closed circle indicate OTC and open triangle indicates wind. Plots marked with different letters differ significantly according to t-tests (*p* < 0.05). Linear approximations are indicated by solid lines for OTC and dashed lines for Wind.

The node count results are shown in **Figure 4A**. Significant differences were observed from June 28 to August 30, September 27 to October 4, and October 18 to November 1, with the node numbers tending to be higher in the OTC group during the cultivation period. Finally, the OTC group had 24.8 ± 0.24 (mean ± SE) nodes, and the Wind group had 23.9 ± 0.22 nodes, indicating that the OTC treatment significantly increased the number of nodes.

Leaf area results are shown in **Figure 4B**. Measurements of leaf area to budding showed significant differences between July and August 12–19, with the leaf area tending to be larger in the OTC group during the cultivation period. Both groups displayed the maximum leaf area during the growing season when measured on August 26, with 56.53 ± 1.64 cm² for the OTC group and 54.40 ± 2.01 cm² for the Wind group. However, no significant differences were observed between groups.

The stem length results are shown in **Figure 4C**. The final stem length measurements showed that that of the OTC group was 1,696.54 ± 21.04 mm and that of the Wind group was 1,323.78 ± 22.48 mm, indicating that the OTC group was significantly taller than the Wind group.

Stem diameter at the base is shown in **Figure 4D**. The final results of stem diameter measurements showed that that of the OTC group was 9.09 ± 0.11 mm and that of the Wind group was 9.55 ± 0.12 mm, indicating that the stems of the Wind group was significantly thicker than the OTC group.

Growth rate results are shown in **Figure 4E**. The growth rate was significantly higher in the OTC group from June 21 to August 23. Subsequently, the growth rates were similar until September 20; however, from September 20 to 27, the growth rate was significantly higher in the wind group. After September 27, the growth rates of both groups decreased but were significantly higher in the OTC group.

**Figure 5:**
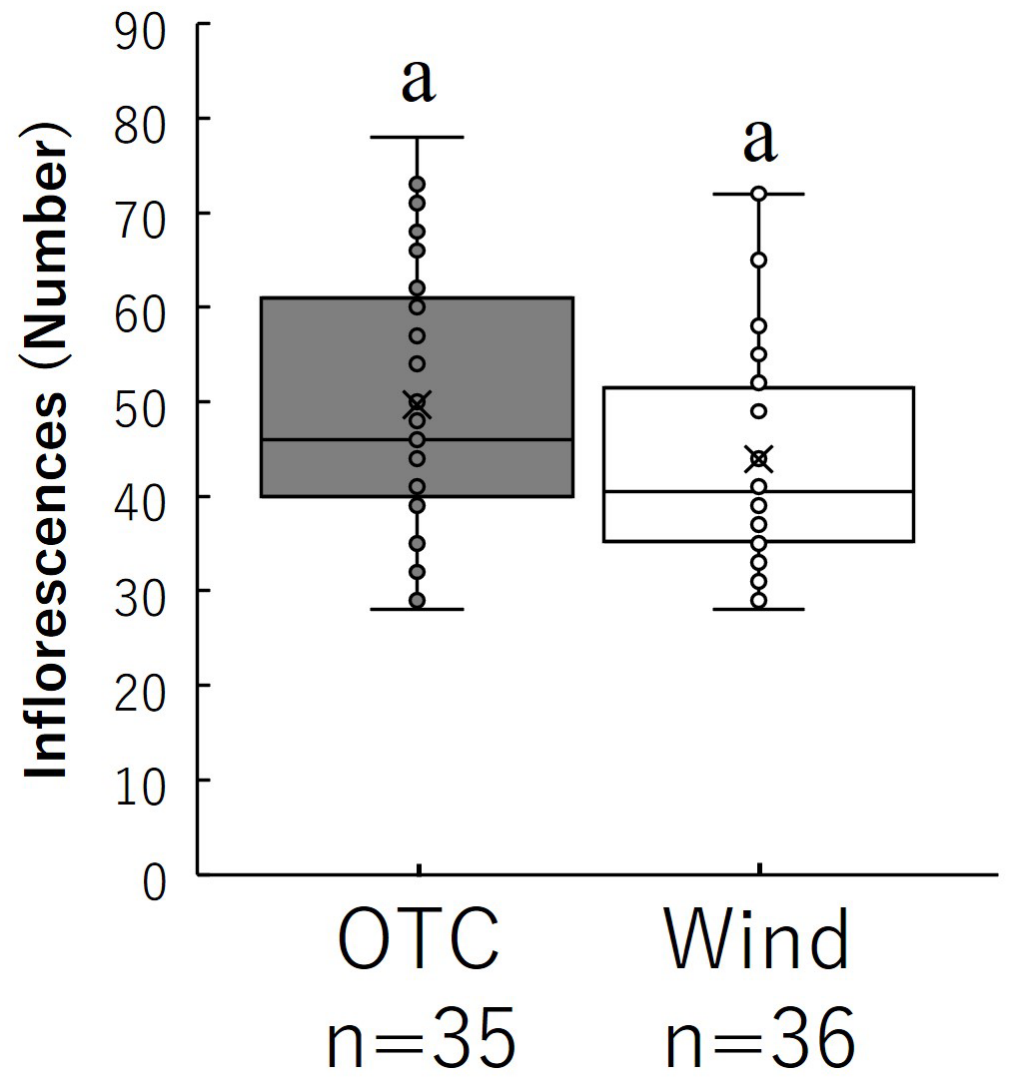
Comparison of the number of inflorescences during growth. Box whisker plots marked with identical letters were not significantly different according to t-test (*p* < 0.05). Crosses indicates position of the mean. **Table 1** shows the average values.

The number of inflorescences per individual is shown in **Figure 5**. Finally, the total number of inflorescences per individual in the OTC group was 49.69 ± 2.83, while the total number of inflorescences per individual in the wind group was 43.94 ± 1.96, but no significant difference was observed.

**Figure 6:**
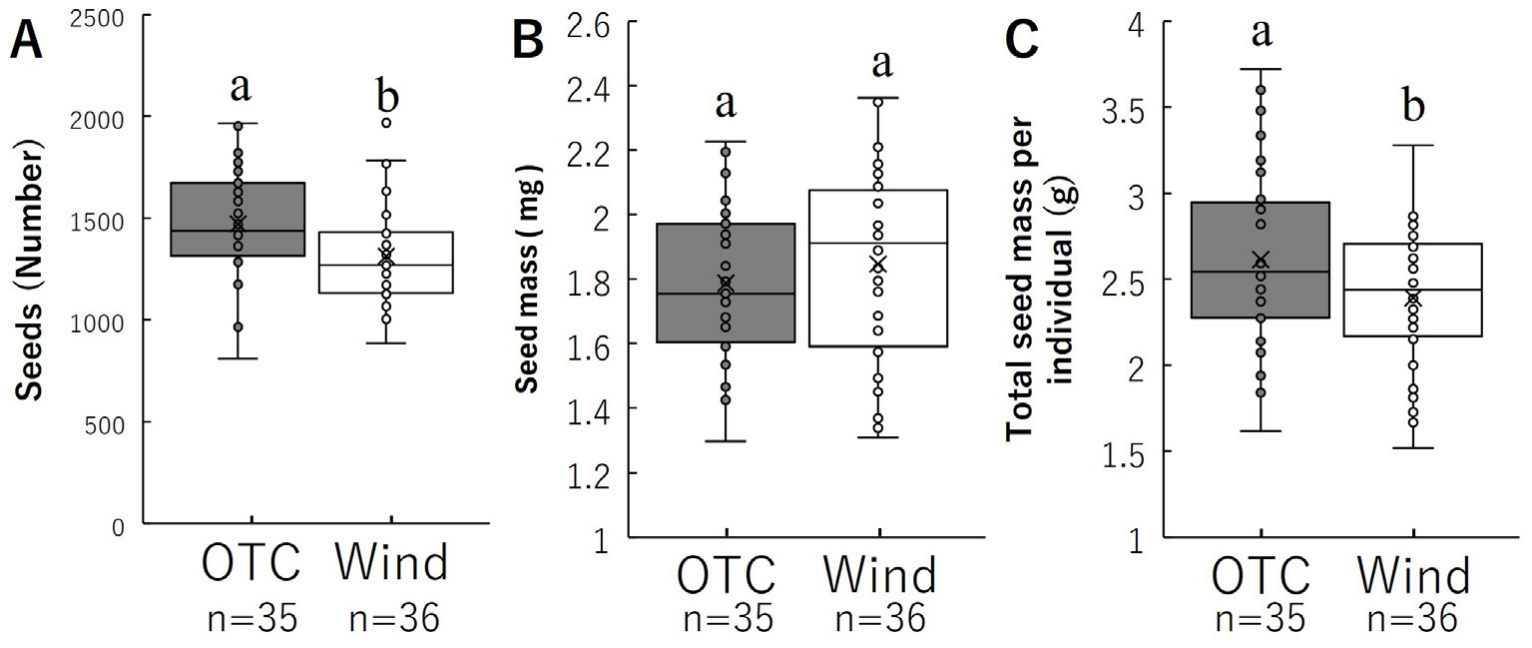
Comparison of number of seeds **(A)** and average seed mass **(B)**, total seed mass per individual **(C)**. Box whisker plots marked with different letters differ significantly according to t-test (*p* < 0.05). Crosses indicates position of the mean. **Table 1** shows the average values.

The number of seeds per individual and average seed mass per seed are shown in **Figure 6** and **Table 1**. The number of seeds per individual was significantly higher in the OTC group than in the WT-treated group (**Figure 6A**). Average seed mass per seed was not significantly different between the two groups (**Figure 6B**). Total seed mass per individual was significantly higher in the OTC group than in the Wind group (**Figure 6C**). These results indicate that the OTC group had a significantly higher total seed mass per individual because of the higher number of seeds.

**Table 1:** Measurements (mean ± standard error) of inflorescences, seed and biomass.

	OTC		Wind	
inflorescences (Number)	55.69 ± 2.11	a	50.86 ± 2.9	a
seeds (Number)	1,472 ± 44.01	a	1.312 ± 40.28	b
seed mass (mg)	1.79 ± 0.00	a	1.85 ± 0.00	a
seed mass per individual (g)	2.61 ± 0.09	a	2.39 ± 0.07	b
biomass				
roots (g)	9.05 ± 0.49	a	8.99 ± 0.21	a
stems (g)	12.15 ± 0.40	a	9.00 ± 0.21	b
leaves (g)	4.01 ± 0.10	a	3.74 ± 0.06	b
above/under ground ratio (-)	1.90 ± 0.07	a	1.52 ± 0.07	b

Columns marked by different letters differ significantly according to the t-test (p<0.05).

### 3.2 Biomass and allocation rates for each organ

**Figure 7:**
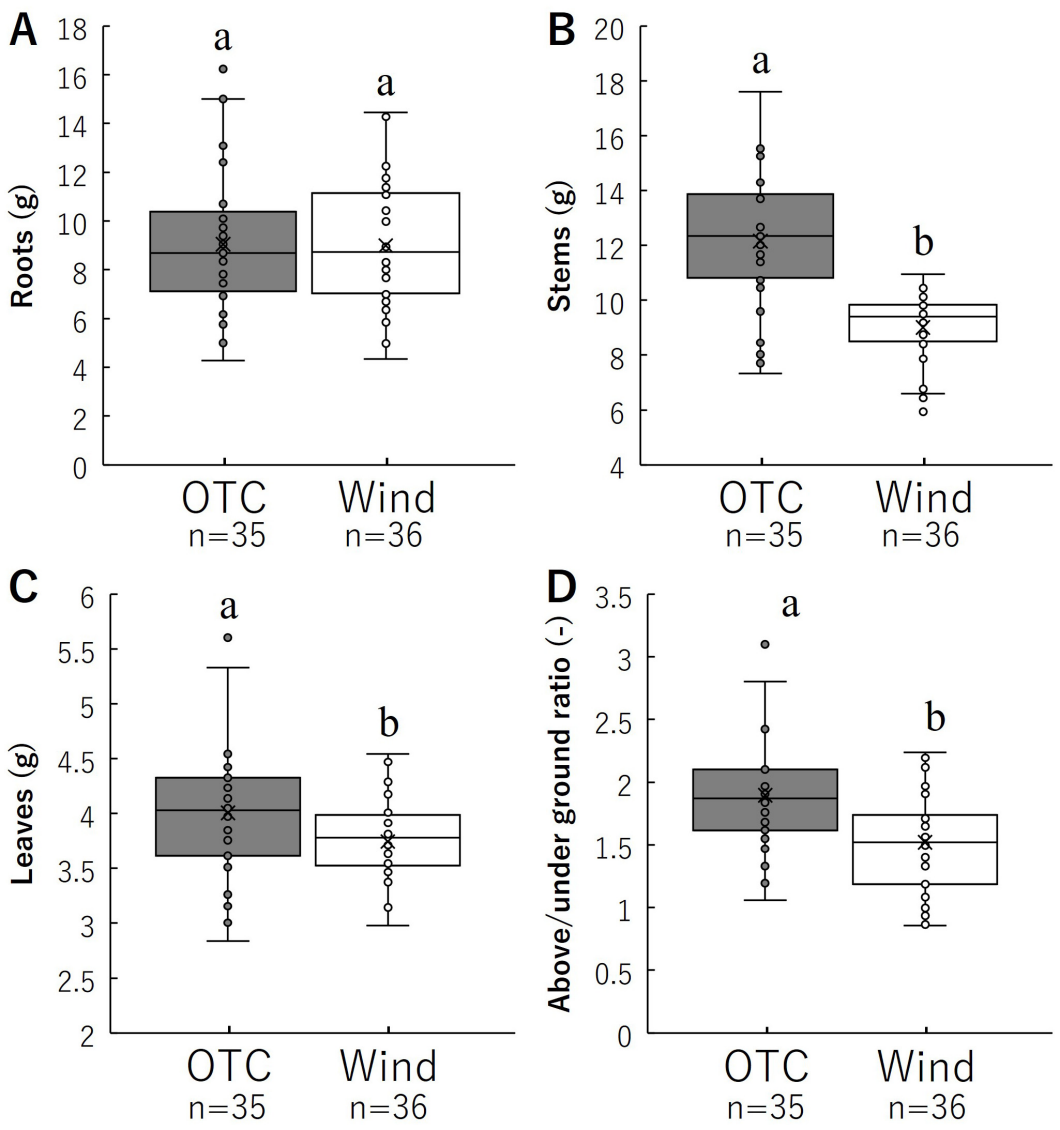
Comparison of roots **(A)** and stems **(B)**, leaves **(C)**, above/under ground ratio **(D)** for biomass. Box whisker plots marked with different letters differ significantly according to t-test (*p* < 0.05). **Table 1** shows the average values.

The dry mass results for each organ are shown in **Figure 7** and **Table 1**. There was no significant difference in the root dry mass per individual between the two groups. However, the dry masses of stems and leaves per individual were significantly higher in the OTC group than in the wind group. Aboveground (stem and leaf) to belowground (root) dry mass ratios are shown in **Figure 7D**. The aboveground and underground dry mass ratios were significantly higher in the OTC group than in the wind group. These results indicate that the OTC group invested significantly more in aboveground areas than the wind group.

The biomass allocation results are presented in **Table 2**. A comparison of the biomass allocation rates within the OTC group showed that the rates of investment in roots, stems, leaves, and seeds differed significantly, with the highest rates of investment in stems. Within the Wind treatment group, significantly different investment rates were observed for leaves and seeds than for roots and stems.

**Table 2:** Measurements (mean ± standard error) of biomass allocation rates to each organ and seeds.

Biomass allocation rates (%)	Roots		Stems		Leaves		Seeds	
OTC	31.91 ± 0.36	b	43.81 ± 0.63	a	14.67 ± 0.83	c	9.60 ± 0.33	d
Wind	36.64 ± 1.05	a	37.49 ± 0.68	a	15.76 ± 0.40	b	10.11 ± 0.37	c

Columns marked by different letters differ significantly according to the Tukey–Kramer test (p<0.05).

## 4 Discussion

Plants adapt to various environmental stressors by acquiring characteristics that allow them to avoid or tolerate them (e.g., Shiba and Fukuda, 2024). Wind is an environmental factor that has various effects on plants. Plant responses to wind typically include reduced photosynthesis (Clark et al., 2000; Burgess et al., 2016), decreased leaf area (Anten et al., 2010; Shiba et al., 2023), inhibition of stem elongation, and an increase in stem diameter to mechanically stabilize the plant (Coutand et al., 2000; Anten et al., 2010; Bang et al., 2010; Shiba et al., 2024b). Understanding the responses of each plant organ to external resources and the environment is central to studying plant diversification. Plant fitness is an important means of evaluating plant responses to environmental factors (Buonaiuto and Wolkovich, 2021; Creux et al., 2021) and serves as an index for evaluating the adaptability of plant characteristics to specific environments and the contribution of individual plants to the production of the next generation (Strauss, 1997). As the resources needed for the growth of roots and leaves, flowering, fruiting, and seed production are not the same, plant investment in these activities is not fixed (Ansaldi et al., 2018; Rusman et al., 2020), and resources are finite, resulting in life history trade-offs in plants (Li et al., 2015). Our results showed that the root mass of *B. pilosa* was not significantly different between the OTC and Wind treatments; however, the allocation ratio was significantly higher in the latter (**Figures 7A, D**; **Table 2**). Wind also improves plant anchorage by strengthening root development (Danjon et al., 2005; Tamasi et al., 2005; Štofko and Kodrík, 2008), and some studies have indicated that wind affects root growth and biomass allocation to roots (Cleugh et al., 1998; Poorter et al., 2012; Gardiner et al., 2016; Feng et al., 2019). Plant anchorage in the soil generally depends on the root system architecture, and the wind group of *B. pilosa* can acquire resistance to wind by allocating resources to the roots. Even the underground parts of *B. pilosa* were shown to be very different between the Wind and OTC groups, and this species also had a large morphological change in each aboveground organ compared to the roots. However, as *B. pilosa* is an annual herb with no significant difference in root mass (**Figure 7A**), it cannot be expected to actively establish itself in areas with extreme winds and will have difficulty growing in environments where it cannot resist this root mass.

Our results indicated that the size and number of leaves in the OTC group of *B. pilosa* were significantly larger and higher, respectively, than those in the wind group (**Figures 4A, B**). Regarding the relationship between wind and leaf size, Shiba et al. (2023) indicated that the leaf size of *Farfugium japonicum* (L.) Kitam. var. *japonicum* (Asteraceae) decreased with increasing wind speed based on wind speed data from the Automated Meteorological Data Acquisition System (AMeDAS) of the Japan Meteorological Agency. In this study, the decreased leaf size in the wind group was a strategy to minimize wind stress and avoid stem lodging, which leads to the physical collapse of photosynthetic capacity and can occur spontaneously owing to the mechanical instability of the stem structure. Therefore, the number of leaves in the wind group decreased to reduce the stress placed on the stem by the wind. In addition, our results indicated that the wind group had significantly shorter stems with increased diameters than the OTC group (**Figures 4C, D**), indicating that *B. pilosa* in the wind group was modified to gain resistance to strong winds. These results indicate the general trends in the effects of wind on plants. In addition, our growth results showed an increase in the stem diameter in the wind group after leaf dwarfing. With respect to these morphological differences, both stem and leaf resources were significantly lower in the Wind group, suggesting that wind stress hindered investment in stems and leaves (**Figures 7B, C**; **Table 1**). What is the relationship between the increase in the proportion of roots and the decrease in stem length, leaf area, and stem number in the wind group? Plants use many trade-off strategies for resource acquisition and distribution to achieve optimal fitness. A comparison of the allocation ratio between the belowground (roots) and aboveground (leaves and stems) parts of *B. pilosa* indicated a trade-off relationship (**Figure 7D**). In particular, the OTC group had the highest investment in stems in terms of resource investment ratio, while the Wind group had similar rates of investment in stems and roots (**Table 2**). This suggests that wind increased the resource allocation ratio of roots and decreased the resource allocation ratio of stems, resulting in individuals in the Wind group having increased resistance and avoidance to wind by reducing the moment on the stem. In addition, the Wind group has shown low leaf biomass and enlarged stems (**Figures 4D**, **7C**), suggesting that resistance and avoidance of wind loads is also demonstrated in the above-ground morphology other than low stem length. Was the high investment in leaves during vegetative growth linked to the high assimilation of this plant, which affected reproductive growth in the OTC group of *B. Pilosa*, such as flowers and seeds? This is discussed as follows.

Floral display is fundamental to plant fitness and can affect pollinator visitation rate and total seed production (Bell, 1985). A common feature of plant reproduction is the production of many more flowers than mature fruits (Stephenson, 1981; Sutherland and Delph, 1984; Sutherland, 1986a, b). Our results indicated that the number of inflorescences did not differ significantly between the OTC and Wind groups (**Figure 5**), suggesting that *B. pilosa* had a similar number of inflorescences regardless of vegetative growth. How can small wind groups produce many inflorescences during vegetative growth? Our results showed that the wind treatment group experienced a rapid acceleration in stem elongation when petioles and inflorescences began to emerge (**Figure 4E**). As the peduncle of *B. pilosa* emerges from nodes with leaves, the wind group could have exhibited rapid stem elongation to increase the number of nodes to form many peduncles. The pool allocated to floral function is limited, and there should be some trade-offs among the behaviors in floral displays (Worley and Barrett, 2000; Caruso, 2004). For example, there are trade-offs in reproductive allocation between flower size and number (Sargent et al., 2007), fruit mass, and seed number for optimal fitness (Sun et al., 2011; Atlan et al., 2015). Our results indicate that the amount of resources allocated to seeds was greater in the OTC group than in the wind group, but the resource allocation ratio in the latter was higher than in the former (**Table 2**). However, there was no significant difference in the number of inflorescences between groups. Why was the Wind group able to form as many inflorescences as the OTC group despite having fewer leaves for photosynthate acquisition? Although the answer to this question could not be determined from the results of this study, *B. pilosa* is often seen on leaves, even in the cold winter months in Japan. Therefore, this species seems to continue to invest the resources acquired through photosynthesis into seed production, even during that period.

The number and size of seeds are important evolutionary traits that affect the reproductive outcomes of many plant species and have been found to vary between and within plant species (Leishman et al., 1995; Silvertown and Bullock, 2003; Moles and Westoby, 2006). The number of seeds is the most direct measure of fitness (Thomson and Hadfield, 2017), and seed size directly influences germination time, germination percentage, and seedling vigor and indirectly determines plant distribution and abundance across different habitats (van Mölken et al., 2005; Silveira et al., 2012). Therefore, it is important to determine the effects of decreased wind stress on *B. pilosa*. The effects of wind stress on seed production have been studied using several methods. Our results showed that the number of seeds in the OTC treatment was significantly higher than that in the wind treatment (**Figure 6A**); however, the mass per seed was not significantly different between the OTC and wind treatments (**Figure 6B**). These results showed that both groups had similar seeds, but the OTC group produced more seeds than the wind group. This significantly affected the formation of offspring, suggesting that the OTC group could produce more offspring than the wind group of *B. pilosa*. The question arose as to why seed number increased in OTC but seed size remained unchanged. It has been reported that increases in weight per seed in animal-adhesive seeds such as *B. pilosa* often have the negative effect on seed dispersal (Kiviniemi and Telenius, 1998). Recently, Qiu et al. (2022) indicated that seed number could vary by more than ten orders of magnitude between different environments. In addition, seeds of *B. pilosa* already have high germination and growth rates easily anywhere (Chauhan et al., 2019), so increasing seed size, which would have a positive effect on germination rates, was not necessary for this species. Considering these, *B. pilosa* had increased only seed number without changing the weight per seed in order to maintain dispersal for successful widespread distribution.

The OTC group may have had many seeds because they were able to increase the amount of resource investment in leaves; that is, adaptive changes in leaves and roots to avoid and/or resist wind stress caused the wind group to decrease the amount of resource investment in leaves, which was reflected in the number of seeds. Many studies have reported that plants adapt to avoid environmental stress by changing the morphological and anatomical traits of their leaves in various environments (Yamada et al., 2011; Hayakawa et al., 2012; Ohga et al., 2012a, b, 2013; Ueda et al., 2012; Yokoyama et al., 2012; Kumekawa et al., 2013; Matsui et al., 2013; Sunami et al., 2013; Shiba et al., 2021, 2022a, 2022b, 2022c, 2024a; Takizawa et al., 2022, 2023). These changes in leaves are related to photosynthetic efficiency; therefore, adaptation processes in these environments may also be involved in reducing the number of seeds. Disc florets of *B. pilosa* have self-compatibility, but insects such as hymenopterans and lepidopterans frequently visit and pollinate this species (Grombone-Guaratini et al., 2004; Budumajji and Solomon-Raju, 2018). However, wind significantly influences flying insects and is an important factor in the spatiotemporal heterogeneity of pollinator flights, influencing insect dispersal and movement. For example, Lewis and Smith (1969) reported that installing windbreaks in orchards tripled the number of insects visiting, and Koga and Namai (2001) revealed the influence of wind on insect pollination of carrot (*Daucus carota* L.) plants. Wind currents are also highly variable and have been reported to affect the stability of pollinator flight and flower landing (Combes and Dudley, 2009; Ravi et al., 2013; Chang et al., 2016). In particular, Chang et al. (2016) indicated that the entry path of honeybees to flowers shifted from multiple directions in still air to one direction in the wind, regardless of flower orientation. In addition, honeybees attempting to land in stronger headwinds did not decelerate smoothly but maintained high flight speeds until contact, resulting in higher peak decelerations upon impact (Chang et al., 2016). These studies showed that wind had a strong influence on the behavior of pollinators and that the decrease in the number of seeds in the wind-affected population of *B. pilosa* may also be related to the influence of wind on the behavior of pollinators. Recently, Budumajji and Solomon-Raju (2018) reported that most foraging visits to the flowers of *B. pilosa* were by butterflies and bees, although wasps and honeybees were also involved in foraging visits. In the future, it will be necessary to compare the number of visits between the OTC and Wind populations of *B. pilosa* to determine the effects of wind on pollinators.

The reproduction of invasive plants in urban areas is an important new area of research that will improve the prediction and management of further spread. Urban environments support the invasive species *B. pilosa*, which ranks among the worst 100 species on the Invasive Alien Species List in Japan. Such mechanisms are likely widespread because *B. pilosa* is often highly abundant in urban systems. Thus, *B. pilosa* and other invasive alien species can exploit urban environments and invest in traits that enhance their reproductive success. The Ministry of the Environment has designated 233 taxa in Asteraceae as invasive species, six species other than *B. pilosa* (i.e., *B. aristosa* (Michx.) Britton, *B. aurea* (Aiton) Sherff, *B. bipinnata* L., *B. frondosa* L., *B. laevis* (L.) Britton, Sterns et Poggenb., and *B. parviflora* Willd.) has been designated as an invasive species in Japan. Future studies should synthesize and use the results of this study as a basis for enhancing the colonization, dispersal, and ecological effects on reproduction.

## Data Availability

The datasets presented in this study can be found in online repositories. The names of the repository/repositories and accession number(s) can be found in the article/**Supplementary Material**.
