# Supplementary material for: Decrease in wind stress leads to an increase in the above ground morphology and number of seeds of an invasive alien species, Bidens pilosa (Asteraceae)
Source: Front Plant Sci. 2024 Nov 8;15:1445437. doi: 10.3389/fpls.2024.1445437 (PMC11581870; doi:10.3389/fpls.2024.1445437)
Supplement: Supplementary file 1 [file Table1.docx]

Supplementary Material

Decrease in wind stress leads to an increase in the number of seeds of an invasive alien species, *Bidens pilosa* (Asteraceae)

# Masayuki Shiba^*^, Nagisa Kobayashi, Shiori Harada, Tatsuya Fukuda

**^*^ Correspondence: Masayuki Shiba: msykshiba48@gmail.com**

# Supplementary Tables

Supplementary Table 1. Datasheet for wind speed (m/s).

Supplementary Table 2. Datasheet for number of nodes. Shaded lines mean no data.

Supplementary Table 2. (Continued)

Supplementary Table 3. Datasheet for leaf area (cm^2^). The data is the average leaf area of the top three leaves.

Supplementary Table 3. (Continued)

Supplementary Table 4. Datasheet for stem length (cm). Shaded lines mean no data.

Supplementary Table 4. (Continued)


Supplementary Table 5. Datasheet for stem diameter (mm). Shaded lines mean no data.

Supplementary Table 5. (Continued)

Supplementary Table 6. Datasheet for growth rate (cm/days). Shaded lines mean no data.

Supplementary Table 6. (Continued)

Supplementary Table 7. Datasheet for number of inflorescences. Shaded lines mean no data. The red box is the data used in Figure 5.


Supplementary Table 7. (Continued)

Supplementary Table 8. Datasheet for number of seeds. Shaded lines mean no data.


Supplementary Table 9. Datasheet for seed mass (mg). Shaded lines mean no data.

Supplementary Table 10. Datasheet for total seed mass per individual (g). Shaded lines mean no data.

Supplementary Table 11. Datasheet for biomass (g). Shaded lines mean no data.
